# High Vascular Tone of Mouse Femoral Arteries *In Vivo* Is Determined by Sympathetic Nerve Activity Via α_1A_- and α_1D_-Adrenoceptor Subtypes

**DOI:** 10.1371/journal.pone.0065969

**Published:** 2013-06-12

**Authors:** Joseph Zacharia, Joseph R. H. Mauban, Hema Raina, Steven A. Fisher, Withrow G. Wier

**Affiliations:** 1 University of Maryland, School of Medicine, Baltimore, Maryland, United States of America; University of Padua, Italy

## Abstract

**Background and purpose:**

Determining the role of vascular receptors in vivo is difficult and not readily accomplished by systemic application of antagonists or genetic manipulations. Here we used intravital microscopy to measure the contributions of sympathetic receptors, particularly α_1_-adrenoceptor subtypes, to contractile activation of femoral artery in vivo.

**Experimental approach:**

Diameter and intracellular calcium ([Ca^2+^]_i_) in femoral arteries were determined by intravital fluorescence microscopy in mice expressing a Myosin Light Chain Kinase (MLCK) based calcium-calmodulin biosensor. Pharmacological agents were applied locally to the femoral artery to determine the contributions of vascular receptors to tonic contraction and [Ca^2+^]_i,_.

**Key results:**

In the anesthetized animal, femoral arteries were constricted to a diameter equal to 54% of their passive diameter (i.e. tone = 46%). Of this total basal tone, 16% was blocked by RS79948 (0.1 µM) and thus attributable to α_2_-adrenoceptors. A further 46% was blocked by prazosin (0.1 µM) and thus attributable to α_1_-adrenoceptors. Blockade of P_2X_ and NPY_1_ receptors with suramin (0.5 mM) and BIBP3226 (1.0 µM) respectively, reduced tone by a further 22%, leaving 16% of basal tone unaffected at these concentrations of antagonists. Application of RS100329 (α_1A_-selective antagonist) and BMY7378 (α_1D_-selective) decreased tone by 29% and 26%, respectively, and reduced [Ca^2+^]_i_. Chloroethylclonidine (1 µM preferential for α_1B_-) had no effect. Abolition of sympathetic nerve activity (hexamethonium, i.p.) reduced basal tone by 90%.

**Conclusion and Implications:**

Tone of mouse femoral arteries in vivo is almost entirely sympathetic in origin. Activation of α_1A_- and α_1D_-adrenoceptors elevates [Ca^2+^]_i_ and accounts for at least 55% of the tone.

## Introduction

The sympathetic nervous system (SNS) plays a major role in maintaining arterial blood pressure, through its effects on the heart, blood vessels, kidneys and adrenal glands. In rats, total block of autonomic ganglionic transmission results in a rapid fall in arterial blood pressure, due acutely to a decrease in total peripheral vascular resistance [1]. Pathologically, SNS hyperactivity is involved in heart failure, hypertension, and metabolic syndrome [2], [3]. Sympathetic nerves release three neurotransmitters onto arterial smooth muscle; noradrenaline (NA), ATP, and neuropeptide Y (NPY). Each binds to several types of pre-and post-junctional receptors that activate several distinct intracellular signaling pathways [4]. The physiological role of each receptor type in a particular blood vessel *in vivo* is difficult to determine however; receptors are present in different amounts in different blood vessels, the amount of SNA varies, and relative amounts of NA, ATP and NPY released vary with the frequency and pattern of nerve action potentials [5], [6]. Here, we sought to define the roles of SNA and of the α_1_-adrenoceptor subtypes in particular, to maintenance of vascular tone in femoral arteries *in vivo*. α_1_-adrenoceptors are classified into 3 functional subtypes α_1A_, α_1B_-, and α_1D_-, corresponding to the cloned α_1_-adrenoceptors, α_1a_-,α_1b_- and α_1d_- respectively [7], [8]. α_1_-Adrenoceptors with low affinity for prazosin (pA_2_<9) have also been identified in functional studies and classified as the α_1L_-subtype [9]–[11]. The α_1L_-adrenoceptor subtype has not been defined and evidence suggests that it is not a separate gene product but a low-affinity state of the α_1A_-adrenoceptor [12], [13]. Many studies have shown the relative importance of these subtypes in the maintenance of arterial blood pressure [14]–[17] or in response to exogenously introduced agonists and/or electrical nerve stimulation in mouse arterial beds *in vitro* [16], [18]–[22]. Nevertheless, these studies have not shown directly the relative importance of the vascular α_1_-adrenoceptor subtypes in vascular tone *in vivo*. Systemic application of receptor blockers or agonists or genetic ablation of receptor subtypes inevitably involve receptors located elsewhere than the vasculature. Similarly, many of the factors potentially influencing vascular tone *in vivo* will be lost when arteries are removed from the animal for study.

In the present study therefore, we utilized a new experimental model, the exMLCK optical biosensor mouse [23]–[26] to determine for the first time the functional roles of the α_1_-adrenoceptor subtypes in basal-state tone of femoral arteries of anesthetized mice *in vivo*. These mice express a FRET-based genetically encoded Ca^2+^/Calmodulin biosensor molecule, based on smooth muscle myosin light chain kinase (MLCK) specifically on smooth muscle cells. The fluorescence in smooth muscle cells of the artery walls provides 1) quantitative measurement of smooth muscle cell [Ca^2+^], and 2) a precise measurement of artery diameter. The preparation we developed [26] provides the ability to apply receptor blockers and agonists locally, to a segment of artery, and thus isolate effects to vascular receptors only in that region of the artery. We have shown previously that systemic effects are avoided through the use of this approach [25], [26]. In this study we determined the functional contributions of α_1_-adrenoceptor subtype to femoral artery tone *in vivo* by using the α_1_-adrenoceptor selective antagonist prazosin [27], the α_1A_-adrenoceptor selective antagonist RS100329 [28], the α_1D_-adrenoceptor selective antagonist BMY7378 [29], [30] and the preferential α_1B_-adrenoceptor alkylating agent, chloroethylclonidine [31].

## Methods

All experiments were approved by the Institutional Animal Care and Use Committee of the University of Maryland, School of Medicine, MD. The transgenic mouse line (ICR, inbred Charles River) was the same as used previously [23]–[26], that expresses a MLCK biosensor that monitors the binding of Ca^2+^-calmodulin through changes in FRET (Forster Resonance Energy Transfer) between cyan (CFP) and yellow (YFP) fluorescent proteins. All mice were maintained on 12∶12-h light/dark schedule at 22–25°C and 45–65% humidity and fed *ad libitum* on a standard rodent diet and tap water. A total of 29 (12 male, 17 female) mice were used, ages of 16–20 weeks, weights 28–32 grams.

### Preparation of Mice

Anesthesia was induced with 1–5% isoflurane (Baxter Pharmaceutical Products Inc., Deerfield, IL) in O_2_. During the surgical procedure and the subsequent experiment anesthesia was maintained with 1.5% isoflurane in O_2_. After induction of anesthesia, mice were placed in a supine position on a custom made temperature-controlled platform set to maintain core temperature of animals at 37–38°C.

### Preparation of Arteries for Recording in vivo

Hair from the hind limb region was removed using a depilatory agent. Under microscopic observation, the femoral artery was exposed via a cutaneous incision in the upper thigh. The underlying connective tissue above the artery was lightly dissected, taking care to avoid severing nerves. After exposing the femoral artery in this way, the animal was moved to the stage of a fluorescence microscope and superfusion of the artery was begun, with the standard physiological salt solution containing (PSS, in mmol/l) 112.0 NaCl, 25.7 NaHCO_3_, 4.9 KCl, 2.0 CaCl_2_, 2.0 MgSO_4_, 1.2 KHPO_4_, 11.5 glucose, and 10.0 HEPES (pH 7.4, equilibrated with gas of 12% O_2_, 5% CO_2_, 83% N_2_). Solutions containing elevated KCl were made by replacing the NaCl with KCl on an equimolar basis. Experiments in which a “zero”-calcium solution was used, the solution had the same composition as the standard PSS with the omission of CaCl_2_ and the addition of Na_2_EGTA (2 mM). Superfusion was at 2 ml/min, 35°C monitored continuously by a temperature probe (Sensortek, Clifton, NJ). The arteries were continuously exposed to antagonist for 10 minutes before arterial diameter was recorded using fluorescent edges of the blood vessel. For CEC treatment, arterial segments were incubated with CEC for 10 min at 35°C followed by washing in PSS for 30 min [19], [32]. For recording of arterial blood pressure, methods were as described previously [26] a small incision is made in the femoral artery and a mouse femoral artery catheter (PE-10) (Braintree Scientific, Braintree, MA) was inserted into the artery. The catheter was connected to a fluid-filled pressure transducer (SP 844, Memscap, Skoppum, Norway). Arterial diameter was recorded from the femoral artery of the other leg. Arterial BP measurements were sampled at 1 kHz with a PowerLab data acquisition system and Lab-Chart Pro (ADInstruments, Colorado Springs, CO). Systemic administration of the autonomic ganglion blocker, hexamethonium, was by intra-peritoneal (i.p) injection (30 µg/g body weight).

### Fluorescence Recording

For imaging femoral arteries *in vivo*, an Olympus MVX10 MacroView microscope (Olympus America, Center Valley, PA) (objective lens: 2X Plan Apochromat, 0.5 NA) was used. Excitation illumination was via a xenon arc lamp (Lambda LS, Sutter Instrument, Novato, CA). For measurements of arterial diameter, the biosensor was excited at 426–446 nm. Emission was collected at 455–485 nm (CFP) and 520–550 nm (YFP) with a charge-coupled device (ORCA ER, Hamamatsu, Bridgewater, NJ). Total optical zoom was set such that an effective imaging of 1.0–2.0 µm/pixel was established. Acquisition of images was set at 1.0 Hz. The microscope was equipped with an image splitter equipped with the appropriate filters for CFP/YFP FRET microscopy (DualView, Photometrics, AZ) and a sensitive CCD video camera (ORCA ER, Hamamatsu Photonics, K.K., Japan). The camera was controlled and images acquired using HCImage (Hamamatsu, Japan).

### Myography

#### Wire myography

Arterial segments of 2 mm length (normalized internal diameter, IC_0.9_, *c.* 310 µm) were mounted in a four-channel wire myograph (Danish Myotech, Aarhus, Denmark) for isometric tension measurement and were maintained in gassed PSS at 35°C. After incubation for 1 hr, the vessels were then normalized, that is, the resting tension–internal circumference (IC) relation was determined for each vessel segment [19], [33], [34]. The resting tension was set to a normal IC of IC_0.9_, where IC_0.9_ = _0.9_IC_100_ and IC_100_ is the internal circumference of the vessel under an effective resting transmural pressure (ERTP) of 100 mmHg (13.3 kPa). ERTP was calculated from the Laplace equation (ERTP = wall tension/(IC/2π)). Lab View software was used for acquisition. At 30 min after normalization, the vessels were exposed to 60 mM KCl solution twice followed by 10 µM noradrenaline in the presence of 60 mM KCl solution. The arteries were considered viable if the equivalent transmural pressure produced by 60 mM KCl was >100 mmHg (13.3 kPa) [32], [35]. Vessels were allowed to equilibrate for a further 30 min before beginning experiments.

#### Functional studies using bath applied phenylephrine in vitro (isolated femoral arteries)

After equilibration, three to four concentration–response curves (CRC) to PE were obtained in each femoral arterial segment (30 min between each CRC). Preliminary experiments showed no significant time-dependent changes in sensitivity. The first CRC was taken as control and subsequent curves were obtained after incubating the vessels with increasing concentrations of the same antagonist for 30 min. To characterize α_1_-adrenoceptors in femoral arteries, RS79948 (0.1 µM, α_2_-adrenoceptor blocker), desmethylimipramine (50 nM, neuronal uptake blocker) and corticosterone acetate (3 µM, non-neuronal uptake inhibitor) were added to the PSS before each CRC. Results are expressed as mean±s.e.m., n being the number of animals.

#### Calcium calibration

Third order mesenteric arterial segments from exMLCK FRET biosensor mice, 2–3 mm in length were dissected [24]. Arteries were mounted on a single channel wire myograph similar to the one described above. Calcium calibration was done on isolated blood vessels because even if a segment of artery could be permeabilized successfully *in vivo*, there is no way to clamp the [Ca^2+^]_i_ concentration reliably due to blood flow. For calcium calibration curves arteries were allowed to stabilize in Krebs solution, followed by incubation of the vessel in a high relaxing (HR) solution for 10–20 min before permeabilization. The high relaxing (HR) and pCa solutions were used during and following permeabilization of the mesenteric vessels were designed to maintain a desired free Ca^2+^ concentration, using a Ca^2+^-EGTA buffering system. The composition of the solutions was calculated with the MAXC Computer Program for calculating free Ca^2+^ concentrations. The HR solution (pCa >9) was composed of the following chemicals (in mM): 53.28 KCl, 6.81 MgCl_2_, 0.025 CaCl_2_, 10.0 EGTA, 5.4 Na_2_ATP, and 12.0 creatine phosphate. The composition of the pCa 4.5 solution was similar to HR, except for the following differences (in mM): 33.74 KCl, 6.48 MgCl_2_, and 9.96 CaCl_2_. The pH of the HR and pCa 4.5 solutions were adjusted to 7.1 with KOH, and ionic strength was held constant (0.15). Solutions containing a desired free Ca^2+^ concentration between pCa 9 and 4.5 were achieved by mixing appropriate volumes of the HR and pCa 4.5 solutions based on the Bathe algorithm. All solutions contained the protease inhibitors leupeptin (1 µg/ml), pepstatin A (2.5 µg/ml), and PMSF (50 µM). Mesenteric arteries were permeabilized with α-toxin from staphylococcus aureus by incubating the vessel segment with 1000 U/ml α-toxin in HR for 1 hour at room temperature. After permeabilization, segments were washed with HR solution and force was allowed to stabilize. The pCa-tension relationship was then determined by bathing the permeabilized vessels in solutions of sequentially increasing Ca^2+^ concentrations, ranging from pCa 8.5 to 4.5, while recording force for 5 min in each solution or until stabilized. The Ca^2+^ induced alteration in tension was expressed as a percent relative to the basal tension at pCa 9. The changes in FRET ratios and force, measured with [Ca^2+^] ranging from 1 nM to 50 µM at excitation wavelength of 426–446 nm, is characterized by a normalized sigmoid curve fit. The calcium calibration graph was plotted with the normalized FRET ratio on the y-axis and calcium concentration on the x-axis. The EC_50_ and Hill Coefficient extracted from the curve were 6.05 (pCa) and 1.4 respectively. R_min_ and R_max_ were calculated by exposing the artery to 0Ca^2+^ (2 mM EGTA and 1 µM acetylcholine) and KCl (60 mM) respectively.

#### Pressure myography

Dissected segments of femoral artery, 1–2 mm in length, were transferred to a recording chamber, where their ends were mounted on glass pipettes (tip diameter 60–100 µm) and secured by 10-0 Ethilon ophthalmic nylon sutures (Ethicon, Somerville, NJ). One pipette was attached to a servo-controlled pressure-regulating device (Living Systems, Burlington, VT), whereas the other was attached to a closed stopcock to study the pressure-dependent effects in the absence of intraluminal flow. The intraluminal pressure was set to 70 mmHg and was continuously superfused with gassed PSS at 35°C. During the entire process, the arteries that developed significant leaks were discarded. Measurements of arterial wall position from transmitted light images were recorded at 2 images/sec with a Nikon ×20 objective.

### Data Analysis

Agonist potency is expressed as the pEC_50_ (the negative logarithm of the concentration required to produce 50% of the maximum response, E_max_). The pEC_50_ and E_max_ values were calculated using the Graphpad Prism software program, which fits CRCs to the four parameter logistic equation below:

Y = Bottom+[top-bottom)/(1+10^(logEC50-X)P^)], where X is the logarithm of the molar concentration of agonist, Y is the response and P is the Hill slope. Antagonist affinity was expressed either as pK_B_ or pIC_50_ values. When three different concentrations of the antagonist were used, pK_B_ values were obtained from the x-intercept of the plot of log (r−1) vs. log(B), where r is the ratio of the agonist EC_50_ in the presence and absence of antagonist and B is the molar concentration of antagonist [36]. If the antagonism met the criteria of competition (Schild slope of unity), then affinity was expressed as pK_B_. When one concentration of antagonist was used to obtain the affinity, estimated pK_B_ values were calculated from the Schild equation [37]: pK_B_ = -log[(B)/(r-1)]. The change in arterial diameter (*in vivo*) was calculated and expressed as a fractional diameter based on full passive diameter with 0[Ca^2+^]. Antagonist potencies were also expressed as mean pIC_50_ values (the negative logarithm of the concentration of antagonist producing 50% inhibition of the prazosin-sensitive component of the vascular tone). Best fit pEC_50_ and E_max_ values obtained from nonlinear regression of CRC (described above) and other mean values were compared by an unpaired t-test for two groups or by repeated measures one-way analysis of variance (ANOVA) followed by Newman-Keuls multiple comparison test (three or more groups) after checking for normality (Kolmogorov–Smirnov test).

#### Calculating [Ca^2+^]_i_

The change in FRET ratios obtained from cumulative addition of antagonist was plotted against the [Ca^2+^] calibration curve to give [Ca^2+^]_i_. Image processing was via custom software, written using Interactive Data Language (IDL) v8.1 (ITT Systems, Inc. USA). To obtain correct FRET ratios with a ‘wide-field’ imaging system, several methodological issues were addressed: 1) spectral overlap, 2) image alignment for ratioing, 3) accounting for ‘background’ fluorescence (i.e. that arising from sources other than the artery being studied, and 4) artery intrinsic fluorescence.

### Drugs Used

The following drugs were used: Phenylephrine, hexamethonium bromide, (−)- noradrenaline bitartrate, acetylcholine chloride, corticosterone acetate, suramin sodium salt, desmethylimipramine, prazosin hydrochloride, α-toxin from staphylococcus aureus, protease inhibitors, creatine phosphate, chloroethylclonidine and (8-[2-[4-(2-methoxyphenyl)-1piperazinyl]ethyl]-8-azaspiro[4.5]decane-7,9-dione (BMY7378) (Sigma, USA). (8aR,12aS,13aS)-5,8,8a,9,10,11,12,12a,13,13a-dechydro-3-methoxy-12-(ethylsulfonyl)-6H-isoquino[2,1-g] [1], [6]naphthyridine hydrochloride (RS79948), 5-Methyl-3-[3-[3-[4-[2-(2,2,2,-trifluroethoxy)phenyl]-1-piperazinyl]propyl]-2,4-(1H,3H)-pyrimidinedione hydrochloride (RS100329), N-[(1R)]-4-[(Aminoiminomethyl)amino -1-[[[(4-hydroxyphenyl)methyl]amino]carbonyl]butyl-α-phenylbenzeneacetamide trifluoroacetate (BIBP3226) (Tocris, USA). Prazosin was dissolved in 30% methanol, hexamethomium in sterile saline for injection, BIBP3226 in DMSO and corticosterone in 20% methanol. Stock solutions of all other drugs were prepared in distilled water. All drug dilutions were made using PSS.

## Results

### Sources of Vascular Tone in Murine Femoral Arteries in vivo

In the anesthetized animal, femoral arteries were constricted to a diameter equal to 54±1% (n = 29) of their passive diameter (PD, in local 0 mM external [Ca^2+^] and 2 mM EGTA). Basal ‘tone’ was thus 46±1% (100%–54%). We found no significant difference in femoral artery tone *in vivo* between female and male mice (16–30 weeks old). Basal tone in females = 46.48±1.49% (n = 15) and males = 46.63±1.25% (n = 11), P>0.05 (un-paired t-test). To determine the component of this tone that might be due to autonomic nervous system activity, we blocked autonomic ganglionic transmission with systemically applied hexamethonium (i.p, 30 µg/gm body weight). This caused a vasodilation of femoral arteries nearly to PD, and a reduction of arterial blood pressure, as we have reported previously [26]. Thus, a major component of the vascular tone of these arteries *in vivo* is revealed as neurogenic. Myogenic tone [38] was absent in these arteries; isolated, pressurized (70 mm Hg) femoral arteries did not develop any vascular tone (data not shown, n = 4) nor myogenic responses to step changes in pressure (30–110 mm Hg).

### Sympathetic Neurotransmitter Receptors

**Figure 1:**
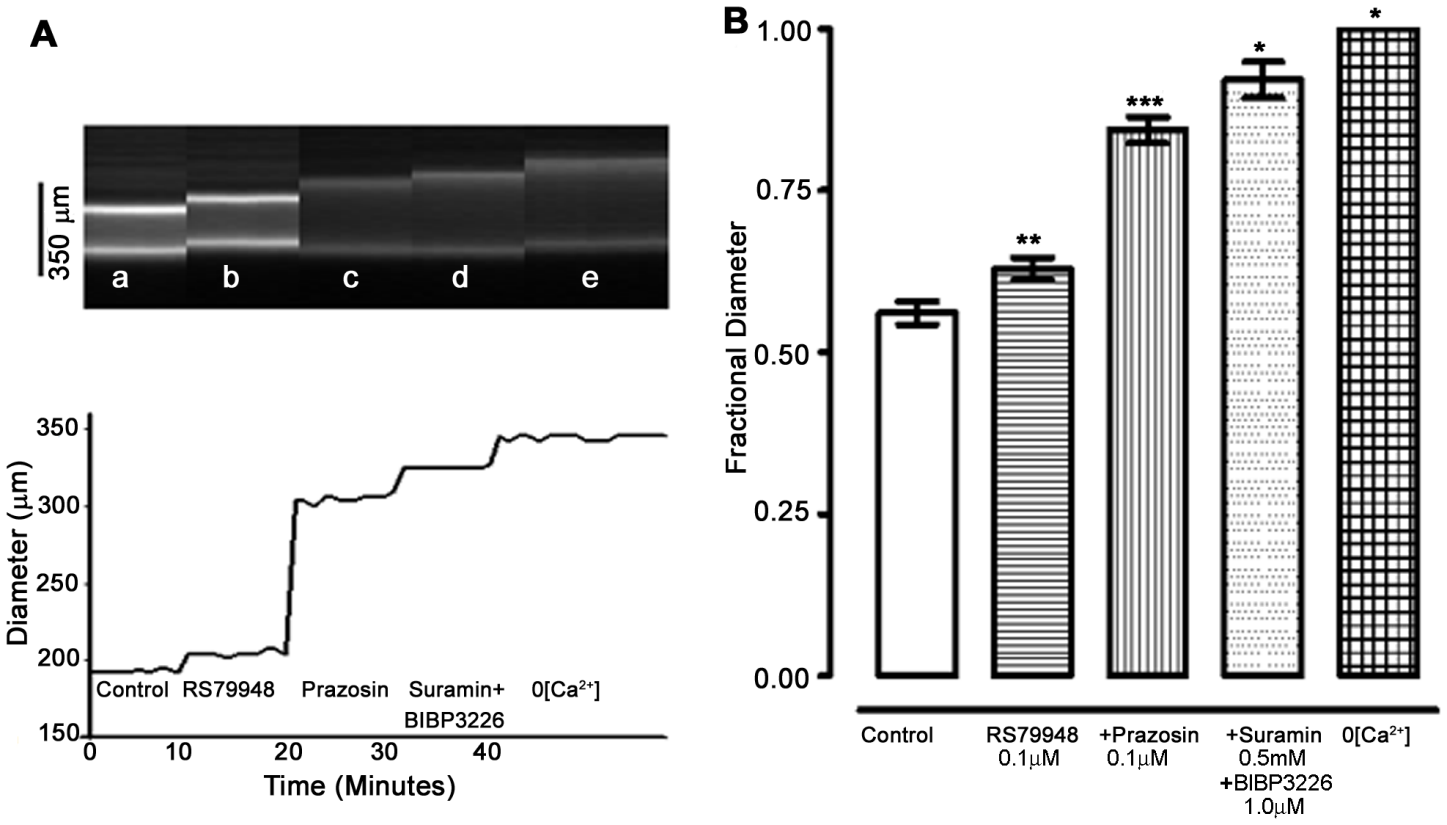
Receptors for sympathetic neurotransmitters activate at least 80% of contractile tone in mouse femoral arteries *in vivo*. (A) Upper panel: 5 (a–e) successive transverse line-scan images of a femoral artery *in vivo.* Artery diameter was obtained as the distance between the two peaks in fluorescence intensity, representing the fluorescence within the walls of the artery. Artery diameter was measured only during the final 10 seconds of a 10 min duration control period or exposure to an antagonist. There is thus a 10 minute gap between each image (a–e) and between each corresponding diameter trace in the lower panel. Lower panel: Artery diameter under each condition (a–e). (a) Control, represents artery in basal state of anesthetized animal. (b–e) Arterial diameter upon on cumulative exposure to RS79948 (0.1 µM) (b), Prazosin (0.1 µM) (c), Suramin (0.5 mM)+BIBP3226 (1 µM) and (e). 0[Ca^2+^]. (B) Average data from 6 arteries. Fractional diameter is the measured diameter divided by the passive diameter for each artery, measured in the presence of 0[Ca^2+^] solution. In the combined presence of α_1_-, α_2_- adrenoceptor, NPY_1_ and P_2X_ receptor blockers, active vascular tone was reduced to less than 20% of the control level. As shown later, α_1_-adrenoceptors are not fully blocked at the concentration of prazosin used (0.1 µM), and thus the tone activated by noradrenaline is greater than shown here. Significance of difference from previous drug treatment, ^*^ *P*<0.05, ^**^ *P*<0.01, ^***^ *P*<0.001 (ANOVA followed by Newman-Keuls multiple comparison test).

The contribution to vascular tone of several neurotransmitter receptors that might be involved in sympathetic neurogenic tone *in vivo* was examined (**Fig. 1**). Local application of RS79948 (0.1 µM, α_2_-adrenoceptor antagonist) and prazosin (0.1 µM, α_1_-adrenoceptor antagonist) reduced vascular tone by 16±3% (n = 12) and 46±4% (n = 9), respectively. Subsequent addition of both BIBP 3226 (1 µM, NPY_1_ blocker) and Suramin (0.5 mM, Purinergic-P_2X_ blocker) also had significant effect on vascular tone (P<0.001, % of tone 22±3, n = 6). A summary of the effects on femoral artery tone *in vivo* of combined block of adrenergic, P_2X,_ and NPY_1_ receptors is shown in **Fig. 1B**. Except for prazosin, the concentrations of all drugs used were maximally effective.

### α_1L_- Adrenoceptors Contribute to Vascular Tone

**Figure 2:**
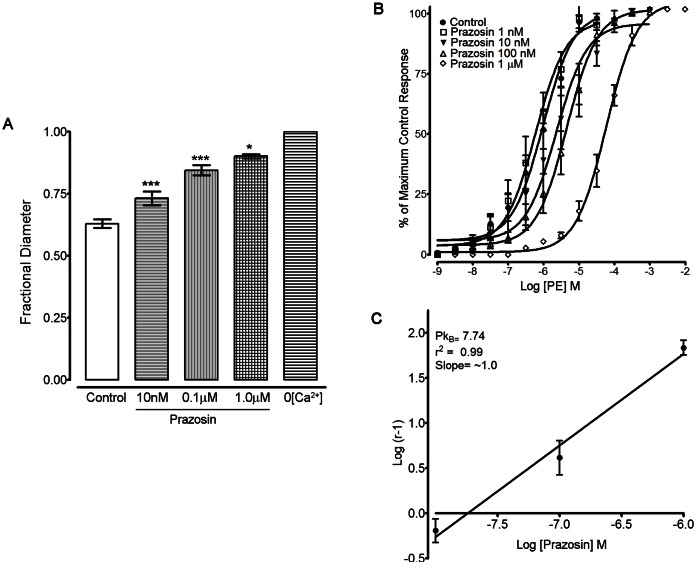
The α_1_-adrenoceptors in the mouse femoral arterial bed are of the α_1L_-subtype. (A) Fractional diameter in femoral arteries *in vivo.* Prazosin (10.0 nM to 1.0 µM) produced successive increases in fractional diameter (i.e. decreased active vascular tone). These arteries were isolated and mounted on a wire myograph for force measurement (B) *Ex vivo* characterization of α_1_-receptors in femoral artery: Prazosin (10 nM–1 µM, n = 5) produced concentration-dependent parallel rightward shifts in the potency of phenylephrine (PE) without significantly affecting the maximum responses. (C) The Schild regression analysis gave a pK_B_ value of 7.74 with a slope of ∼1.0. All experiments were carried in the presence of RS79948 (0.1 µM). Significance of difference from previous drug treatment, ^**^ *P*<0.01, ^***^ *P*<0.001 (ANOVA followed by Newman-Keuls multiple comparison test).

Since pre- and post-junctional α_2_-adrenoceptors activated by neurally released NA could affect the contribution of α_1_-adrenoceptors to vascular tone *in vivo*, the experiments to determine the α_1_-adrenoceptor subtypes involved were all carried out in the presence of RS79948 (0.1 µM). *In vivo*, local prazosin (10 nM-1 µM, n = 8, **Fig. 2A** **, supplementary table. 1**) produced concentration dependent inhibition of vascular tone (pIC_50_ value of 8.0±0.1). To determine the α_1_-adrenoceptor subtype based on affinity to prazosin, femoral arteries were isolated and mounted on a wire myograph for isometric force recording and the effect of prazosin on the concentration-response curves to phenylephrine (PE) was determined. Prazosin (10 nM–1 µM, n = 5, **Fig. 2B**) produced a rightward shift of the concentration response curve; the Schild plot gave a pK_B_ value of 7.74 with a slope of 1.01±0.11, not significantly different from 1.0 (**Fig. 2C**). Based on affinity to prazosin, the α_1_-adrenoceptors in the mouse femoral artery are of α_1L_-subtype.

### α_1A_-_,_ α_1B_-, α_1D_- Adrenoceptor Subtypes

**Figure 3:**
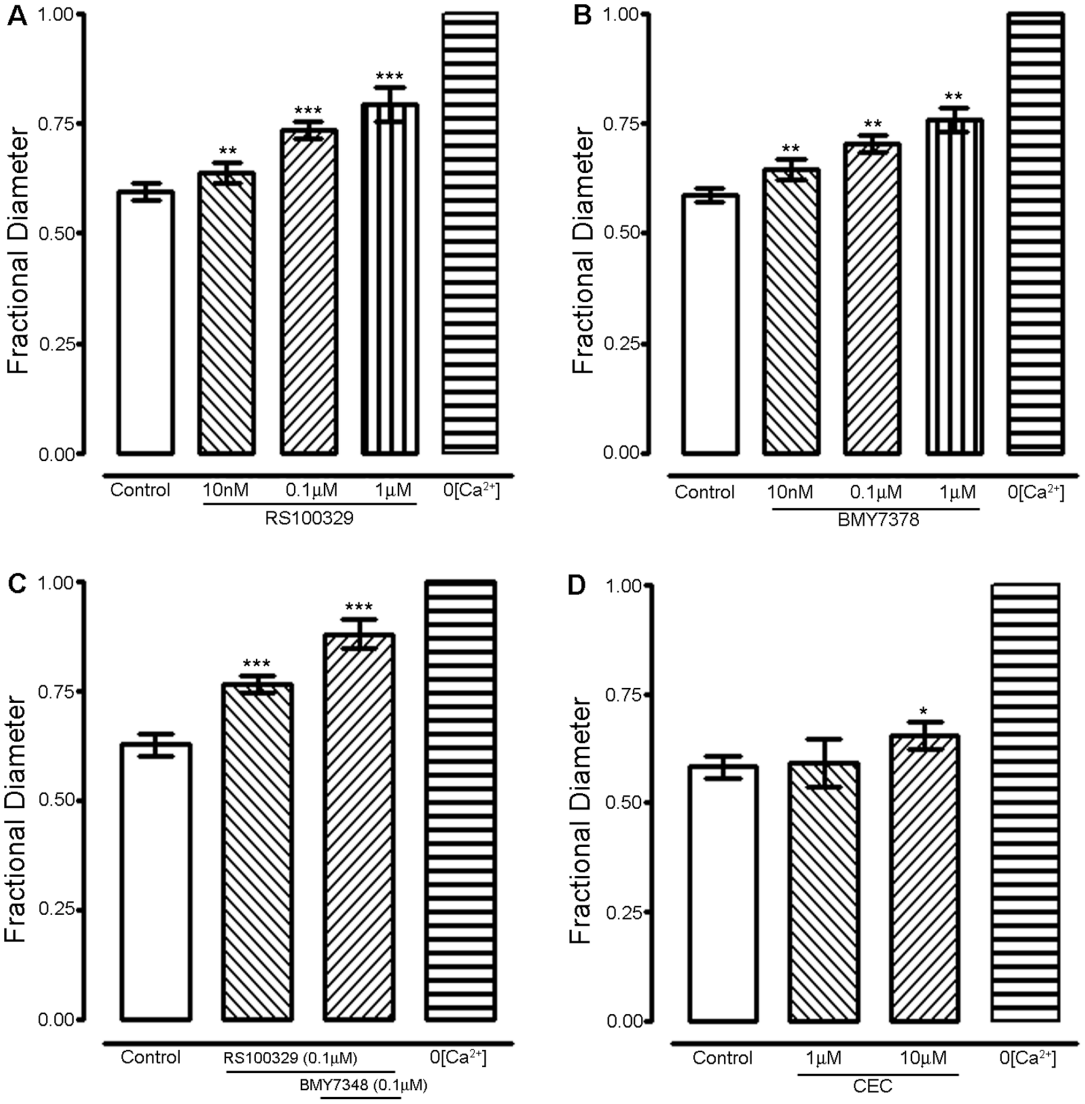
α_1A_- and α_1D_- adrenoceptor subtypes are the major adrenoceptor subtypes that activate vascular tone in femoral artery *in vivo*. All experiments were carried in the presence of the α_2_-adrenoceptor blocker, RS 79948 (0.1 µM), the effects of which are represented by the ‘control’ bars. (A) Effect of RS100329 (10 nM-1 µM, n = 5) and (B) BMY7378 (10 nM-1 µM, n = 5) on active vascular tone of mouse femoral artery. (C) Combined effect of RS 100329(0.1 µM) and BMY7378 (0.1 µM). (D) Effect of alkylating agent CEC (1 and 10 µM, n = 3). Significance of difference from previous drug treatment, ^*^ *P*<0.05, ^**^ *P*<0.01, ^***^ *P*<0.001 (ANOVA followed by Newman-Keuls multiple comparison test).

We examined the contributions of α_1_-adrenoceptors by using subtype specific antagonists. RS 100329, specific for α_1A_-adrenoceptors, (10 nM-1 µM, n = 5, **Fig. 3A** **, supplementary table. 1**) significantly inhibited vascular tone with a pIC_50_ value of 7.4±0.1. The fractional diameter increased by 0.14±0.01 (P<0.001) with 0.1 µM RS 100329. BMY 7378, specific for α_1D_-adrenoceptors (10 nM-1 µM, n = 5, **Fig. 3B** **, supplementary table. 1**) also inhibited vascular tone significantly with a pIC_50_ value of 7.2±0.2 and an increase in fractional diameter by 0.12±0.01 (P<0.001) with 0.1 µM BMY7378. The combination of RS100329 (0.1 µM) and BMY7378 (0.1 µM) effected a change in fractional diameter of 0.25±0.03 (n = 5, **Fig. 3C**) similar to prazosin (0.1 µM, 0.21±0.02 **Fig. 2A**). At 1 µM, CEC, preferentially selective for α_1B_-adrenoceptors, (1 µM, n = 3) had no significant effect on vascular tone, but higher doses of CEC (10 µM, n = 4) did have a small effect, increasing fractional diameter by 0.07±0.01 (P<0.01, **Fig. 3D** **, supplementary table. 1**).

### α_1-_Adrenoceptors and [Ca^2+^]_i_

**Figure 4:**
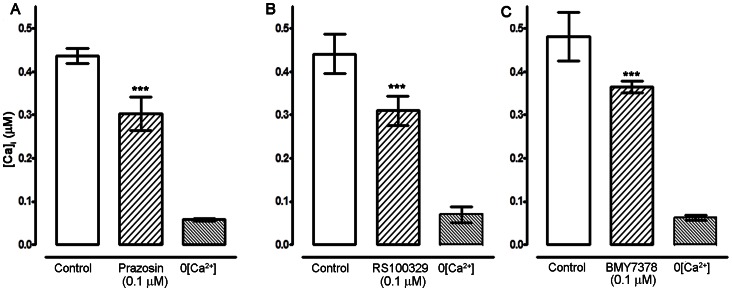
Block of α_1A/1D_-adrenoceptors reduces intracellular [Ca^2+^] in femoral artery *in vivo*. All experiments were carried in the presence of RS 79948 (0.1 µM) represented by the ‘control’ bar. (A) Change in [Ca^2+^]_i_ on local application of prazosin (0.1 µM, n = 8) on mouse femoral artery *in vivo* (n = 8). (B) Decrease in [Ca^2+^]_i_ in the presence of RS100329 (0.1 µM, n = 5) and (C) Decrease in [Ca^2+^]_i_ in the presence of BMY7378 (0.1 µM, n = 5) in mouse femoral artery *in vivo*. Significance of difference from previous drug treatment, ^***^ *P*<0.001 (ANOVA followed by Newman-Keuls multiple comparison test).

The presence of the genetically encoded biosensor in the arterial smooth muscle provided not only a convenient way to measure artery diameter, but also provided a measurement of smooth muscle intracellular [Ca^2+^]. The inhibition of vascular tone using α_1_-adrenoceptor antagonists was also evident in the changes in intracellular calcium calculated by the changes in CFP and YFP ratios in ex-MLCK biosensor animals. Prazosin (0.1 µM) decreased [Ca^2+^]_i_ by 0.18±0.03 µM **(** **Fig. 4A, **P<0.001). RS100329 (0.1 µM) significantly decreased [Ca^2+^]_i_ by 0.15±0.02 µM (**Fig. 4B, **P<0.001). Similarly, BMY7378 significantly reduced [Ca^2+^]_i_ by 0.11±0.03 µM (**Fig. 4C, **P<0.001). CEC (10 µM) did not significantly change [Ca^2+^]_i._ (not shown).

## Discussion and Conclusions

Vasoconstriction (‘tone’) of femoral artery of living anesthetized mice is substantial (∼ 50%) and activated mostly (∼90%) by the sympathetic nervous system, through receptors for NA, ATP and NPY. Our previously published work demonstrated gender differences in myogenic reactivity in cochlear arteries [39] and others have noted gender differences in rat cerebral vessels [40], [41]. However, no significant difference in femoral artery tone *in vivo* was recorded between female and male mice in the present study. Combined block of the adrenoceptors α_2_-, α_1A_-_,_ and α_1D_- reduced vascular tone by ∼71% (Fig. 3C). Additional block of NPY_1_ and P_2X_ receptors typically reduced tone by a further ∼ 22% (of the original amount, Fig. 1B), for a total reduction of ∼90%. As might be predicted therefore, abolition of SNA by block of autonomic ganglion transmission also reduced vasoconstriction to ∼90% of passive diameter. Thus we conclude that at least ∼ 90% of the tone of femoral arteries *in vivo* is attributable to neurally released NA, ATP and NPY. A small (∼10%) component of the femoral artery vasoconstriction did not arise from sympathetic nerve activity. Neither did this component arise from the myogenic mechanism [38], as isolated femoral arteries lacked completely any active response to intra-luminal pressure changes. It seems likely that the remaining component of tone is activated by circulating substances (e.g. Angiotensin II), endothelial vasoconstrictors (e.g. endothelin) and/or many other vasoactive substances present in the normal circulation and arterial wall.

### α_2_-Adrenoceptors

A small but significant contribution to tone from activation of post-synaptic α_2_-adrenoceptors was evident. Post-synaptic α_2_-adrenoceptors are known to play a small but significant role in vasoconstriction in isolated (*ex vivo*) murine tail, first order cremaster, and femoral small arteries [19], [21], [42]. The net effect of α_2_-adrenoceptor inhibition was dilation, in these experiments. We expect the α_2_-adrenoceptor selective inhibitor RS79948 to block both pre and post junctional α_2_-adrenoceptors. A net vasodilation could have resulted if the antagonist produced a greater inhibition of post-junctional α_2_-adrenoceptor mediated contraction than of pre-junctional α_2_-adrenoceptor mediated inhibition of neurotransmitter release.

### α_1L_-Adrenoceptors

α_1_-adrenergic receptors with low affinity for prazosin, *viz.* α_1L_- adrenoceptors, have not been found previously in mouse vasculature. Rather, the high-affinity type, α_1H_-, occurs in mouse first order mesenteric, aorta, carotid, caudal, and femoral small arteries [18], [19], [22]. The pIC_50_ value we measured for the effect of prazosin on femoral arteries *in vivo* was ∼8.0. Clearly however, that value is not a direct measure of affinity for prazosin of the α_1_-adrenoceptors activated by neuronally released NA, since equilibrium conditions do not apply *in vivo*. Under equilibrium conditions of the wire myograph organ bath, we measured a pK_B_ for prazosin antagonism of phenylephrine of 7.74, consistent with the presence of α_1L_-adrenoceptors [9], [11], [12]. α_1L_-adrenoceptor is a pharmacological phenotype of α_1A_-subtype and is derived from the same gene [12], [13], [43]. Although not found previously in mouse, α_1L_-adrenoceptors are present in rat femoral arteries [44], [45], and small mesenteric arteries [46].

### α_1A_-Adrenoceptor

RS100329, a selective α_1A_-adrenoceptor antagonist, inhibited vascular tone in a concentration dependent manner. The pIC_50_ value for RS100329 (7.4) was significantly lower than that of prazosin (8.0). Since RS100329 and prazosin have similar affinity for α_1A_-adrenoceptors, but RS100329 has significantly lower affinity for α_1D_-adrenoceptors than prazosin, this might suggest that not all of the prazosin-sensitive α_1_-adrenoceptors activated by neurogenically released noradrenaline are of the α_1A_-subtype. This possibility was explored with selective α_1D_-adrenoceptor antagonists (below).

### α_1D_-Adrenoceptor

The α_1D_-adrenoceptor antagonist BMY7378 also inhibited vascular tone in a concentration dependent manner. BMY7378 does bind also to α_1A/B_-adrenoceptors, but with lower affinity than it does to α_1D_-adrenoceptor and than prazosin does [29]. On femoral artery, similar to the case with RS100329, the pIC_50_ values of BMY 7378 (7.2) were less than that of prazosin. Thus, the presence of α_1D_-adrenoceptors was confirmed and the existence of other α_1_-adrenoceptor subtype.

### α_1B_-Adrenoceptor

Finally, α_1B_-adrenoceptors appeared to contribute negligibly to femoral vascular tone, since CEC at 1 µM had no effect on vascular tone or [Ca^2+^]_i_. The small effect on vascular tone with CEC at 10 µM that was observe may be attributable to α_1A/D_-adrenoceptors since at that concentration, CEC, also alkylates α_1A/D_-adrenoceptors [47]–[49].

### Summary

When RS100329 (0.1 µM) and BMY7378 (0.1 µM) were used in combination, the prazosin (0.1 µM) sensitive response was completely eliminated. This suggests the major role from α_1A_- and α_1D_-adrenoceptors is maintenance of sympathetic neurogenic tone in mouse femoral artery *in vivo*. Consistent with this, α_1A_-adrenoceptors play a major role in tonic maintenance of mouse blood pressure [14], [50] and vasoconstriction in resistance vasculature [14], [18], [19]. The role of α_1D_-adrenoceptors in mediating nerve mediated responses are well documented in rat caudal artery where the authors suggested that α_1D_-adrenoceptors are restricted to the junctional region by neuronal activity, but if the nerves are lost, these receptor subtypes spread from the postsynaptic region along the smooth muscle [51]. Pharmacological and immuno-histochemical studies have shown the presence of α_1D_-adrenoceptors in rat femoral arteries [52]. α_1D_-adrenoceptors are known to play a modulatory role which is constitutively active in contractile tone in rat conductance arteries [53]. α_1D_-adrenoceptors are also involved in nerve mediated responses in rat [35] and mouse [19] femoral resistance arteries. Studies in α_1D_-adrenoceptor knock out models have directly shown that α_1D_-adrenoceptor participates in the regulation of systemic blood pressure [15]. In conclusion, the present study has shown a dominant role of adrenoceptor subtypes α_1A_- and α_1D_- in neurogenic tone of mouse femoral arteries *in vivo*.

## Supporting Information

Figure S1 (TIF)

Figure S2 (TIF)

Figure S3 (TIF)

Figure S4 (TIF)

Table S1 (DOC)
