# Supplementary figures and images for: High Vascular Tone of Mouse Femoral Arteries In Vivo Is Determined by Sympathetic Nerve Activity Via α1A- and α1D-Adrenoceptor Subtypes
Source: PLoS One. 2013 Jun 12;8(6):e65969. doi: 10.1371/journal.pone.0065969 (PMC3680395; doi:10.1371/journal.pone.0065969)

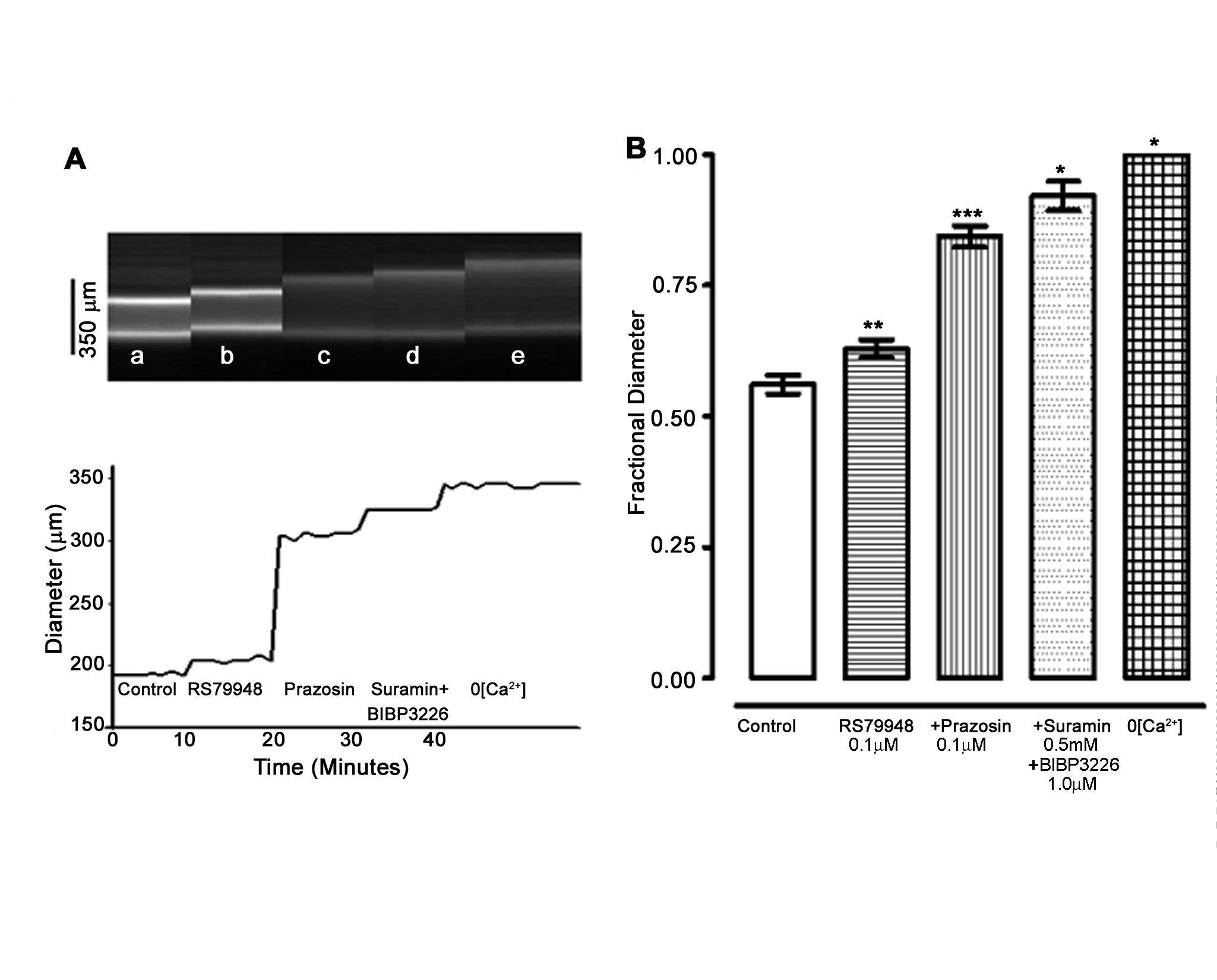

Supplement: Figure S1 — (TIF) [file pone.0065969.s001.tif]

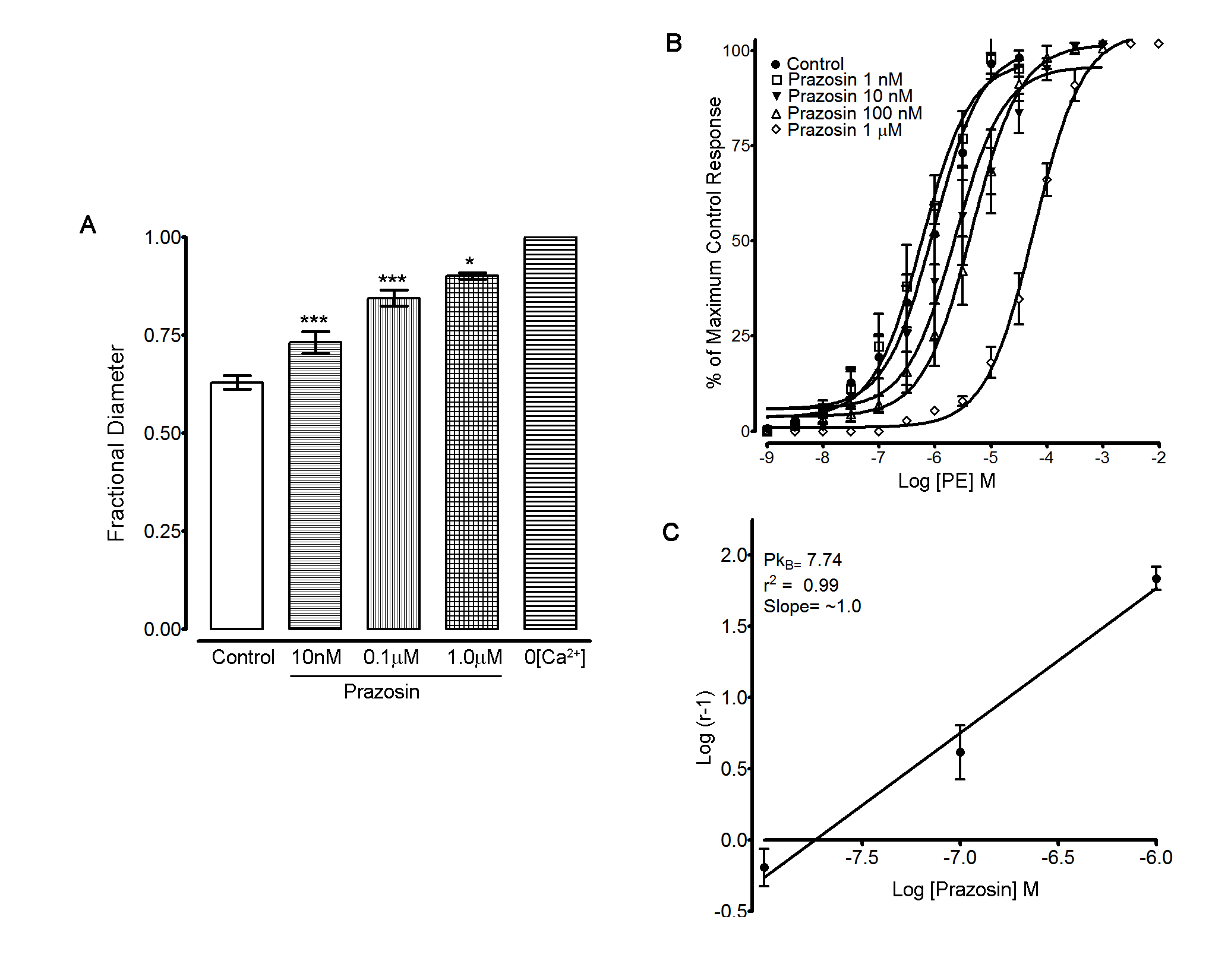

Supplement: Figure S2 — (TIF) [file pone.0065969.s002.tif]

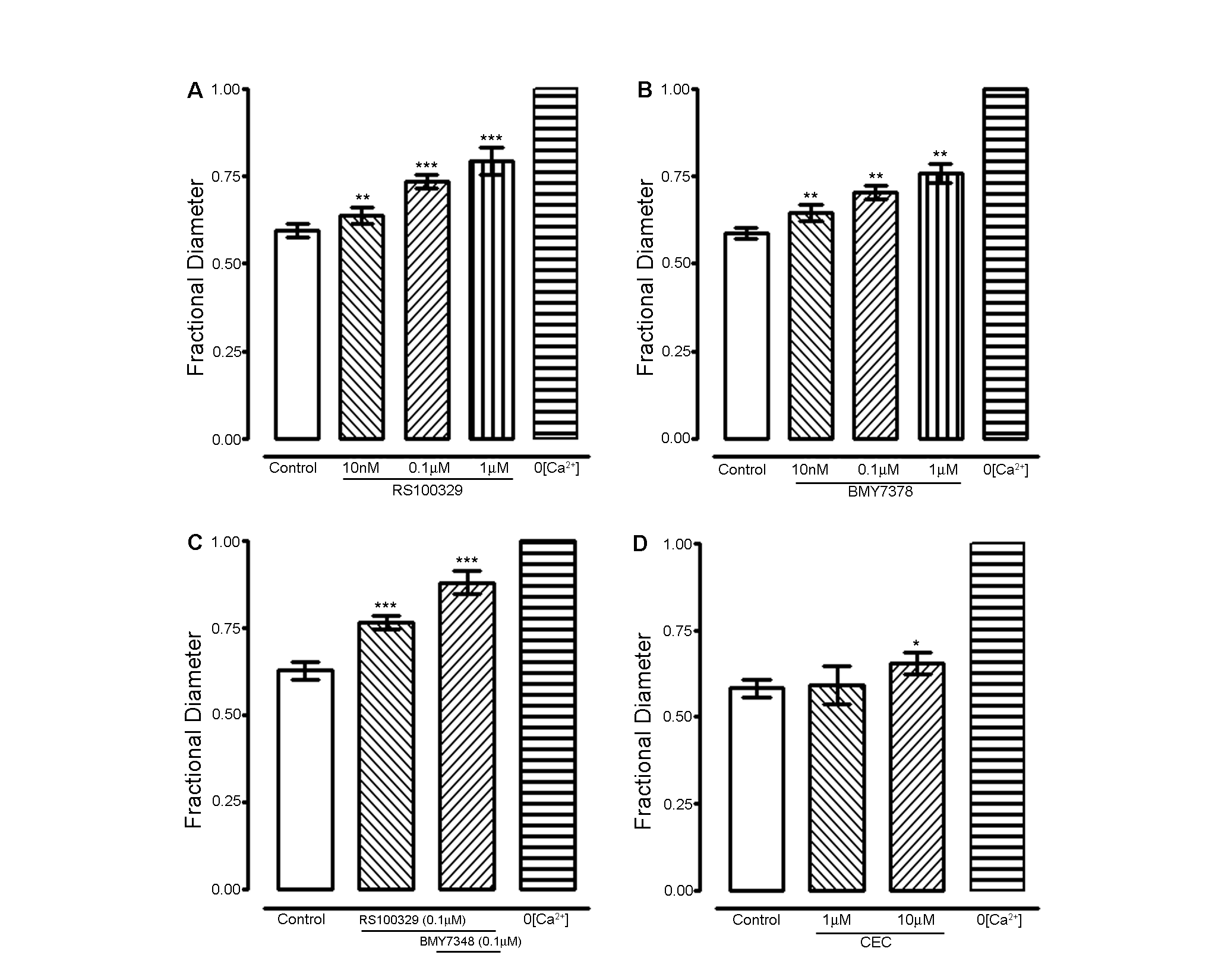

Supplement: Figure S3 — (TIF) [file pone.0065969.s003.tif]

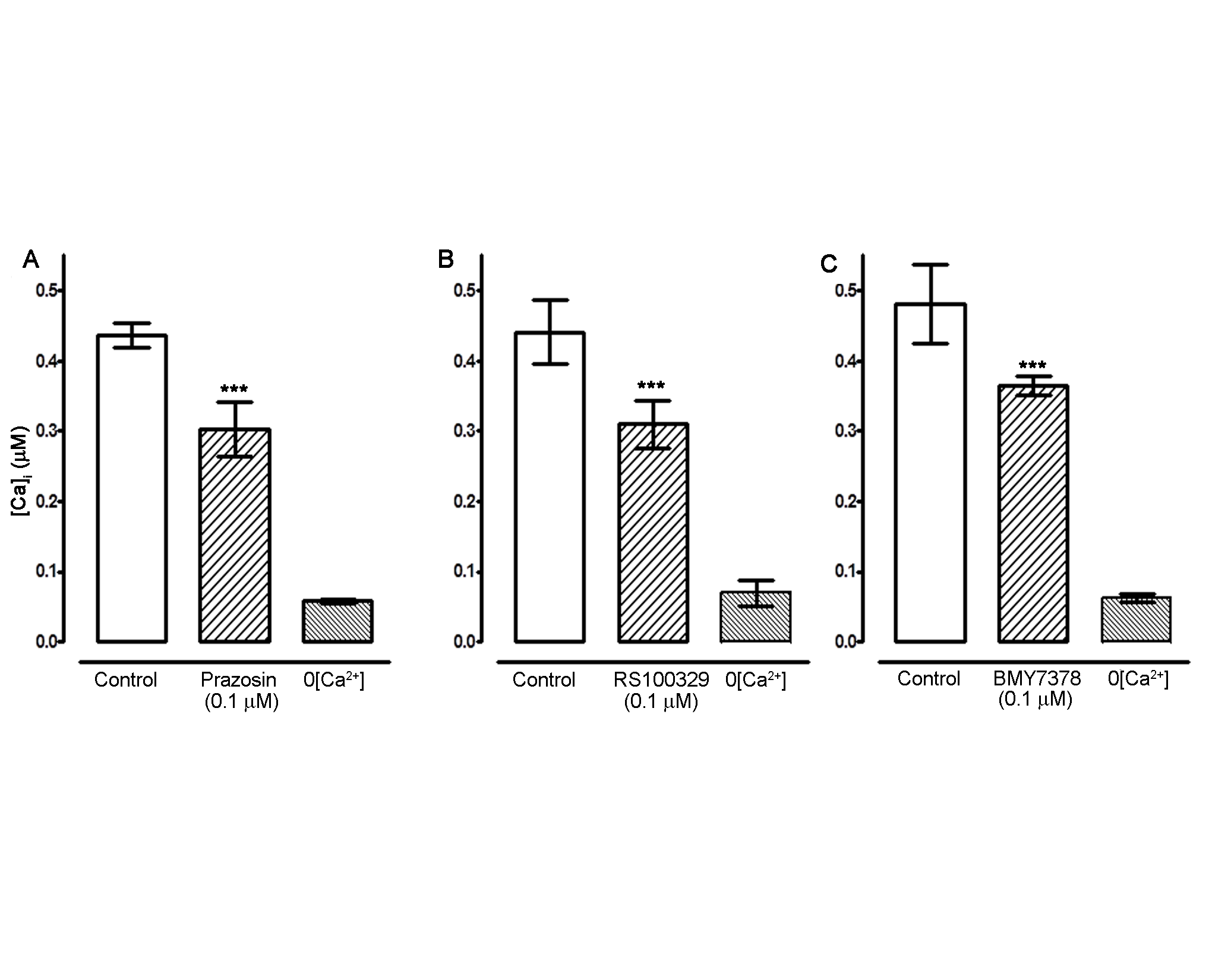

Supplement: Figure S4 — (TIF) [file pone.0065969.s004.tif]
