# Supplementary material for: High Vascular Tone of Mouse Femoral Arteries In Vivo Is Determined by Sympathetic Nerve Activity Via α1A- and α1D-Adrenoceptor Subtypes
Source: PLoS One. 2013 Jun 12;8(6):e65969. doi: 10.1371/journal.pone.0065969 (PMC3680395; doi:10.1371/journal.pone.0065969)
Supplement: Table S1 — (DOC) [file pone.0065969.s005.doc]

**Concentration** (μM) **0** **0.01 0.1 1.0**

**Drug**

Prazosin (n=8) 0.62±0.01 0.73±0.02 0.84±0.02 0.90±0.01

RS100329 (n=5) 0.60±0.02 0.64±0.02 0.740±0.2 0.79±0.02

BMY 7378 (n=5) 0.59±0.03 0.65±0.02 0.70±0.02 0.76±0.03

CEC (n=3) 0.58±0.03 0.60±0.05

Table 1: **Summary of the effects of various α_1_-adrenoceptor antagonists *in vivo* on femoral artery tone.** The data is represented by the fractional diameter (1.00 being passive) of the femoral artery calculated *in vivo*. All experiments were carried in the presence of the α_2_-adrenoceptor blocker, RS 79948 (0.1 μM).
